# A new target for G protein signaling

**DOI:** 10.7554/eLife.31106

**Published:** 2017-09-11

**Authors:** László Csanády

**Affiliations:** 1 Department of Medical BiochemistrySemmelweis UniversityBudapestHungary; 2 MTA-SE Ion Channel Research GroupSemmelweis UniversityBudapestHungary

**Keywords:** TRPM3, G-protein, pain, ion channels, DRG neurons, sensory neurons, Mouse

## Abstract

G protein-coupled receptor stimulation inhibits TRPM3 channel activity through direct binding of the G_βγ_ subunit to the channel.

**Related research article** Badheka D, Yudin Y, Borbiro I, Hartle CM, Yazici A, Mirshahi T, Rohacs T. 2017. Inhibition of transient receptor potential melastatin 3 ion channels by G-protein βγ subunits. *eLife* **6**:e26147. doi: 10.7554/eLife.26147

**Related research article** Quallo T, Alkhatib O, Gentry C, Andersson DA, Bevan S. 2017. G protein βγ subunits inhibit TRPM3 ion channels in sensory neurons. *eLife* **6**:e26138. doi: 10.7554/eLife.26138

**Related research article** Dembla S, Behrendt M, Mohr F, Goecke C, Sondermann J, Schneider FM, Schmidt M, Stab J, Enzeroth R, Leitner MG, Nuñez-Badinez P, Schwenk J, Nürnberg B, Cohen A, Philipp SE, Greffrath W, Bünemann M, Oliver D, Zakharian E, Schmidt M, Oberwinkler J. 2017. Anti-nociceptive action of peripheral mu-opioid receptors by G-beta-gamma protein-mediated inhibition of TRPM3 channels. *eLife* **6**:e26280. doi: 10.7554/eLife.26280

Many of the cells in our body communicate by releasing small molecules that bind to receptors on the surface of target cells. These molecules include hormones and, in the case of nerve cells, neurotransmitters. Signal transduction pathways then relay the information from the receptor to inside the cell and either activate or inhibit ‘effector’ proteins that cause the cells to respond appropriately. Heterotrimeric G proteins – protein complexes that consist of three different subunits named α, β and γ – provide one such pathway, and work with cell surface receptors called G protein-coupled receptors (GPCRs; Figure 1A).

**Figure 1.**
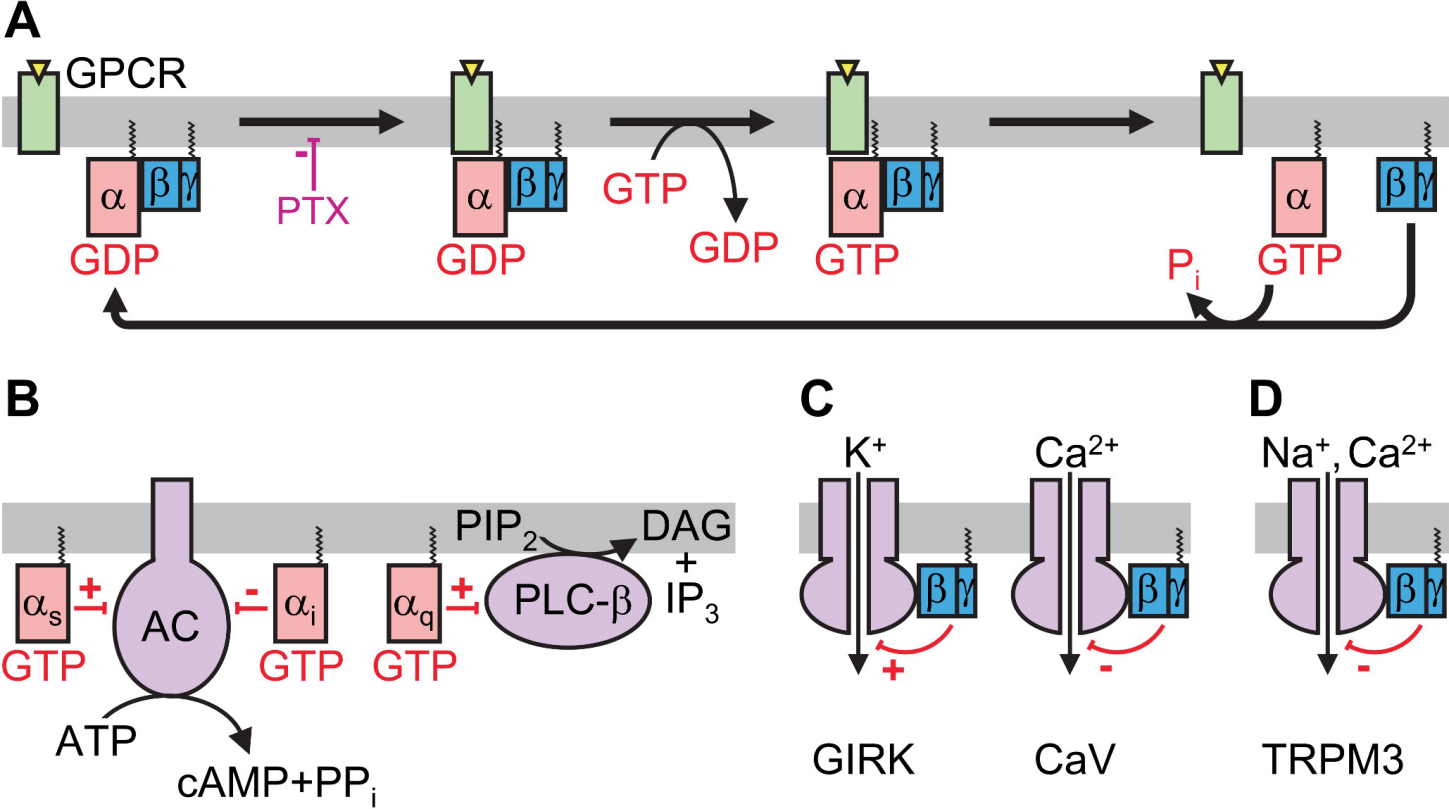
Simplified schematics of heterotrimeric G protein signaling pathways. (**A**) Functional cycle of a heterotrimeric G protein. In the resting state (left), heterotrimeric G proteins consist of α, β and γ subunits bound together. The α subunit is bound to a molecule of GDP, and the complex is anchored to the intracellular surface of the cell membrane. When a G protein-coupled receptor (GPCR) is activated by an agonist molecule binding to its extracellular surface, the heterotrimeric complex can interact with the cytosolic surface of the GPCR. Pertussis toxin (PTX) can inhibit this interaction. The molecule of GDP bound to the α subunit is released and a molecule of GTP binds in its place, causing the heterotrimer to dissociate into G_α_-GTP and G_βγ_ subunits. Upon the breakdown of GTP to form GDP and phosphate (P_i_), the heterotrimer reforms. (**B**) Signal transduction through the G_α_ subunit. Some classes of G_α_ activate (α_s_) or inhibit (α_i_) adenylyl cyclase (AC), the enzyme that produces the second messenger cyclic AMP (cAMP) by removing the terminal pyrophosphate (PP_i_) from ATP. Others (α_q_) activate the enzyme phospholipase C-β (PLC-β), which cleaves an important component of the plasma membrane's inner leaflet called phosphatidylinositol bisphosphate (PIP_2_), converting it into diacylglycerol (DAG) and inositol trisphosphate (IP_3_). (**C**) Signal transduction through the G_βγ_ subunit. G_βγ_ activates G-protein-gated inward rectifier K^+^ (GIRK) channels (left) but inhibits voltage-gated calcium ion channels (CaV; right). (**D**) TRPM3 channels are inhibited by direct binding of G_βγ_.

The structure and mechanism of heterotrimeric G proteins has been studied at atomic resolution (Oldham and Hamm, 2008). In the resting state the α subunit (G_α_) binds to a molecule called GDP and is tightly associated with the β and γ subunits, forming a heterotrimer. When the complex interacts with an activated GPCR, the molecule of GDP is exchanged for GTP, and the G protein complex dissociates into two parts: G_α_-GTP and a stable G_βγ_ dimer. Both G_α_ and G_βγ_ contain ‘anchors’ that keep them attached to the cell membrane, but allow them to diffuse laterally along the membrane to find their target effector proteins. Eventually, G_α_ breaks down the GTP to form GDP, and G_α_-GDP associates with G_βγ_ to reform the heterotrimer.

In the ‘conventional’ mode of signal transduction (Gilman, 1987) G_α_-GTP activates or inhibits a target enzyme, depending on which class of α subunit is involved (Figure 1B). For example, an inhibitory α subunit (G_αi_) inhibits the enzyme that produces a chemical messenger called cyclic AMP (or cAMP). Heterotrimeric G proteins may also regulate ion channels within the membrane via a different pathway. For instance, the GIRK channels (which are responsible for slowing the heart rate) are activated by G_βγ_ directly binding to them (Figure 1C; Logothetis et al., 1987).

Now, in eLife, three groups featuring researchers based at institutes in Germany, the UK, the US and Canada independently present evidence of a new target for GPCR signaling: an ion channel called Transient Receptor Potential Melastatin 3 (TRPM3; Badheka et al., 2017; Quallo et al., 2017; Dembla et al., 2017). Expressed abundantly in sensory neurons, TRPM3 channels help organisms to sense heat and make them more sensitive to pain during inflammation (Vriens et al., 2011). The new reports suggest that the activation of TRPM3 channels is greatly reduced if the channels are also stimulated by any of a variety of GPCRs.

The three studies systematically probed individual steps of the G protein regulatory pathway to dissect its mechanism. First, each of the groups independently show that the inhibition of TRPM3 can be overcome by pre-treatment with pertussis toxin. This toxin prevents the inhibitory G_αi_ subunit from interacting with the activated GPCR, locking the complex in the resting trimeric state (Figure 1A). Thus, TRPM3 inhibition requires the heterotrimeric G protein to dissociate. But is the inhibiting signal carried through G_αi_-GTP or through G_βγ_?

The studies accumulate strong evidence showing that the G_α_ subunit is not involved. Johannes Oberwinkler of Philipps-Universität Marburg and colleagues (including Sandeep Dembla and Marc Behrendt as joint first authors) did not detect any interaction between G_αi_ and TRPM3. Moreover, they and Tibor Rohacs of New Jersey Medical School and co-workers – who include Doreen Badheka and Yevgen Yudin as joint first authors – show that wild-type inhibitory G_αi_ subunits do not decrease the activity of TRPM3, and neither can mutant subunits that cannot break down GTP and are therefore permanently active. Furthermore, Talisia Quallo and colleagues at King’s College London show that GPCR-mediated TRPM3 inhibition is unaffected by an inhibitor that selectively acts upon the inhibitory G_αi_ subunit. Dembla et al. and Badheka et al. also show that altering the concentration of the chemical messenger cAMP (which is decreased by the activity of inhibitory G_αi_ subunits) has no effect on either the activity or inhibition of TRPM3.

On the other hand, all evidence points to a role for G_βγ_ in signaling to TRPM3. Badheka et al. and Dembla et al. both show that TRPM3 activity is strongly inhibited by the overexpression of G_βγ_, whereas the overexpression of engineered proteins that bind to G_βγ_ (and so prevent it from interacting with TRPM3) eliminates GPCR-mediated TRPM3 inhibition. Co-immunoprecipitation experiments demonstrate a direct interaction between G_βγ_ and TRPM3. Finally, Badheka et al. show that the flow of ions through TRPM3 channels is strongly and reversibly inhibited when the cell membrane is flushed with purified G_βγ_, but not with G_α_ or boiled G_βγ_. These elegant studies thus reveal that G_βγ_ inhibits TRPM3 by directly binding to the channel (Figure 1D).

Interesting mechanistic questions remain. Not all G_α_ subunits are inhibitory and it is unclear whether G_βγ_-mediated TRPM3 regulation also occurs with heterotrimers containing other G_α_ subunits. Like GIRK channels, TRPM3 channels require a molecule called PIP_2_ in the membrane in order to open (Badheka et al., 2015; Tóth et al., 2015). Because some other G_α_ subunits (arbitrarily named G_αq_ subunits) lead to the localized depletion of PIP_2_ (Figure 1B), heterotrimers containing these subunits do not activate GIRK (Wang et al., 2014; Figure 1C). However, they should enhance inhibition of TRPM3 through GPCRs. Indeed, artificially expressing TRPM3 in human embryonic kidney cells enabled Badheka et al. to show that TRPM3 is readily inhibited through co-expressed G_αq_-linked receptors even when the concentration of PIP_2_ inside cells is buffered. This demonstrates that G_βγ_ released from G_αq_-containing heterotrimers can also inhibit TRPM3. However it remains to be established whether G_αq_-linked GPCRs (or any other GPCRs for that matter) contribute to this process in any native cell.

Finally, in vivo experiments by all three groups highlight the practical relevance of the uncovered pathway for pain signaling in the peripheral nervous system: molecules that bind to and activate two types of GPCRs – the GABA-B and μ opioid receptors – significantly reduce TRPM3-dependent pain. Of note, the strongest peripheral painkillers currently available activate the μ opioid receptor, but cause severe adverse effects in the brain such as addiction, tolerance or respiratory depression. The new findings suggest that peripheral pain might be better treated by drugs that inhibit TRPM3 directly.
